# Virus Infection Suppresses *Nicotiana benthamiana* Adaptive Phenotypic Plasticity

**DOI:** 10.1371/journal.pone.0017275

**Published:** 2011-02-17

**Authors:** Stéphanie Bedhomme, Santiago F. Elena

**Affiliations:** 1 Instituto de Biología Molecular y Celular de Plantas, Consejo Superior de Investigaciones Científicas-Universidad Politecnica de Valencia, Valencia, Spain; 2 The Santa Fe Institute, Santa Fe, New Mexico, United States of America; University of South Florida College of Medicine, United States of America

## Abstract

Competition and parasitism are two important selective forces that shape life-histories, migration rates and population dynamics. Recently, it has been shown in various pathosystems that parasites can modify intraspecific competition, thus generating an indirect cost of parasitism. Here, we investigated if this phenomenon was present in a plant-potyvirus system using two viruses of different virulence (*Tobacco etch virus* and *Turnip mosaic virus*). Moreover, we asked if parasitism interacted with the shade avoidance syndrome, the plant-specific phenotypic plasticity in response to intraspecific competition. Our results indicate that the modification of intraspecific competition by parasitism is not present in the *Nicotiana benthamiana* – potyvirus system and suggests that this phenomenon is not universal but depends on the peculiarities of each pathosystem. However, whereas the healthy *N. benthamiana* presented a clear shade avoidance syndrome, this phenotypic plasticity totally disappeared when the plants were infected with TEV and TuMV, very likely resulting in a fitness loss and being another form of indirect cost of parasitism. This result suggests that the suppression or the alteration of adaptive phenotypic plasticity might be a component of virulence that is often overlooked.

## Introduction

Competition and parasitism are two important selective forces that shape life-histories, migration rates and population dynamics [1]. For example, populations of *Drosophila melanogaster* evolved at high levels of intraspecific competition present a pattern of adaptation including a higher larval feeding rate, a higher tolerance to urea [2] and a higher growth rate [3]. In plants, intraspecific competition is also known to regulate densities and the sessile characteristic of plants adds another constrain. Indeed, plants are unable to move to look for resources, light or soil minerals, and avoid or reduce the competition from conspecifics. However, numerous species present a “shade avoidance syndrome” when grown at high population densities: they go through a suite of morphogenic changes such as stem elongation, suppression of branching, altered biomass allocation, and accelerated flowering [4]. All these modifications are triggered by a low red to far-red light ratio (R:FR) occurring when a plant is shading another one because leaves absorb more in the red than in the far-red. The change in the R:FR ratio is sensed by the plant photoreceptors, particularly phytochromes, which are the starting point of a signaling cascade leading to the reallocation of resources and changes in the growth pattern [5]. This phenotypic plasticity is likely to improve light harvesting and its adaptive value has been demonstrated in *Impatiens capensis* [6], by manipulating the growth pattern of seedlings using light qualities that induce, or not, the shade avoidance syndrome, organizing the obtained plants in high and low density plots and measuring the fitness of each phenotype in each case. Plants presenting the shade avoidance syndrome because they were induced by low R:FR ratio had a higher fitness than non-induced plants in high density plots and the pattern was reversed in low density plots.

Adaptive and non-adaptive phenotypic plasticity in response to intraspecific competition is not the only factor that can influence life-history traits. Indeed, pathogens are also known to affect life-history traits, in a way that generally results in a fitness reduction. This fitness reduction constitutes the virulence. Infected individuals often present differences in growth patterns and resource consumption and in animals the differences can extend to behavior and motility. Consequently, the presence of infected individuals in a population generates heterogeneity and is likely to affect the intraspecific competition relationship. Indeed, in a partially infected population, three cases of competition can be distinguished: competition between healthy individuals, competition between healthy and infected individuals and competition between infected individuals [7]. If we take into account that the infected category may in itself be heterogeneous because of the presence of pathogen genotypes or species of different virulence and because of variation in the pathogen load, the situation becomes more complex and the interaction between parasitism and intraspecific competition is likely to result in a continuum of competition intensities.

A number of experiments have been conducted to investigate the interaction between intraspecific competition and parasitism. All of them are in the framework of a homogenous infected category. Among the studies involving plants, some use crop [8] or weeds [9], [10] and investigate the competition interactions between plants resistant and sensitive to a pathogen, (used as a biological control agent in the latter case) in the presence and in the absence of the pathogen. In all cases, the results indicate an interaction between the effects of intraspecific competition and parasitism, with the infected plants suffering more from infection if they are in competition with healthy ones than when they are in competition with infected ones. Similar effects have been shown in non-agricultural systems [11], [12]. More recently, using the *Arabidopsis thaliana - Cucumber mosaic virus* pathosystem, it was demonstrated that the additional cost of parasitism due to the modification of intraspecific competition is expressed in terms of virulence or tolerance depending on the *A. thaliana* ecotype infected [13], thus adding another level of complexity to the studied interaction.

In animals, similar indirect costs of parasitism due to the modification of the intraspecific competition relationship has been shown in at least two insect systems [7], [14], but no such effect was found in *Rana pipiens* parasitized by the trematode *Echinostoma trivolvis* [15].

From the data collected until today, the most frequent pattern is one of modification of intraspecific competition relationship by parasitism that results in a higher virulence expressed by individuals in competition with healthy conspecifics. This is likely to translate at the population level in a negative relationship between the prevalence of a pathogen and the virulence expressed by the host it infects. Indeed, at low prevalence, the majority of the intraspecific competition interactions experienced by an infected individual occur with healthy individuals and this results in a high indirect cost of parasitism and high expressed virulence, whereas at high prevalence, the majority of the competition interactions of infected individuals happen with other infected ones, so they pay few indirect cost of parasitism and express lower levels of virulence.

**Figure 1:**
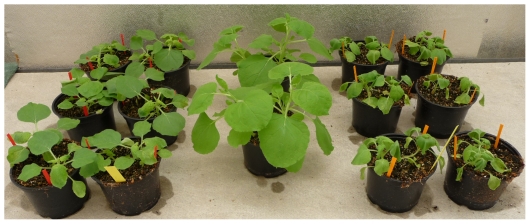
Symptoms of TEV and TuMV on *N. benthamiana.* Plants inoculated with TEV (6 plants on the left) or with TuMV (6 plants on the right) and healthy plants (2 plants in the center). The inoculation took place on four-weeks old plants and the picture was taken 10 dpi.

The present study has two goals: (i) investigate how the indirect cost of parasitism due to the modification of intraspecific competition is affected by the presence in the population of two phylogenetically related pathogens of different virulence and (ii) establish how the “shade avoidance syndrome” - the plant-specific adaptive phenotypic plasticity in response to intraspecific competition - interplays with the effect of parasitism. For this, we used *Nicotiana benthamiana* as a host and *Tobacco etch potyvirus* (TEV) and *Turnip mosaic potyvirus* (TuMV) as pathogens. Both viruses are ssRNA viruses from the *Potyviridae* family and have a moderately wide host range [16]. *N. benthamiana* is a host for the two viruses. In its hosts, TEV induces stunting and mottling, necrotic etching and malformation in leaves; the combination of these symptoms results in a decrease in host fitness [17]. TuMV induces mottling, mosaic, malformation in leaves and wilting. When inoculated at the same dose, TuMV induces stronger symptoms and a stronger reduction of the biomass than TEV (figure 1).

## Materials and Methods

The infectious clone pTEV-7DA (GeneBank DQ986288) was kindly provided by Prof. J.C. Carrington (Oregon State University). This clone contains a full-length cDNA of TEV and a 44 nt long poly-T tail followed by a BglII restriction site cloned into the pGEM-4 vector downstream of the SP6 promoter. A stock of infected tissue was generated before the beginning of the experiment. The plasmid was linearized with BglII; the linearized plasmid was purified with phenol-chloroform (pH 6.7), sodic acetate (3 M, pH 5.5) and ethanol 96%. 5′ capped infectious RNAs were obtained by *in vitro* transcription using SP6 mMESSAGE mMACHINE1 kit (Ambion Inc.) and following the manufacturer's instructions. Viral RNA was diluted in inoculation buffer (100 mg/mL carborundum, 0.5 M K_2_HPO_4_) to a final concentration of 725 ng.µ L^−1^. 28 four-weeks old *N. benthamiana* were inoculated by abrasion with 3 µL by plant. 11 days post-inoculation (dpi), infected tissue was collected, powdered in liquid nitrogen and inoculation buffer was added (0.71 g of infected tissue per mL of inoculation buffer). The viral content of the obtained stock was quantified by inoculation of a dilution series on the local lesion host *Chenopodium quinoa* [18]. It was thus determined that the stock contained 1.25×10^5^ lesion-forming units (LFU) per gram of infected tissue.

The infectious clone pTuMV[L72] (Genebank AF530055.2) was kindly provided by Prof. N.H. Chua (Rockefeller University). This clone contains a full-length cDNA of the isolate YC5 of TuMV cloned in the pCaMVCN downstream of the *Cauliflower mosaic virus* (CaMV) 35S promotor. As for TEV, a large stock of infected tissue was generated before the beginning of the experiment. A culture of *Escherichia coli* containing the pTuMV[L72] plasmid was realized in 1 L of LB containing 100 µg/mL of ampicilin. Plasmids were extracted and 28 four-weeks old *N. benthamiana* were inoculated by abrasion with the plasmid solution (250 µg/plant). From the infected tissue obtained, the same procedure as for TEV was followed and the obtained stock was evaluated to contain 3.75×10^4^ LFU per gram of infected tissue.

**Figure 2:**
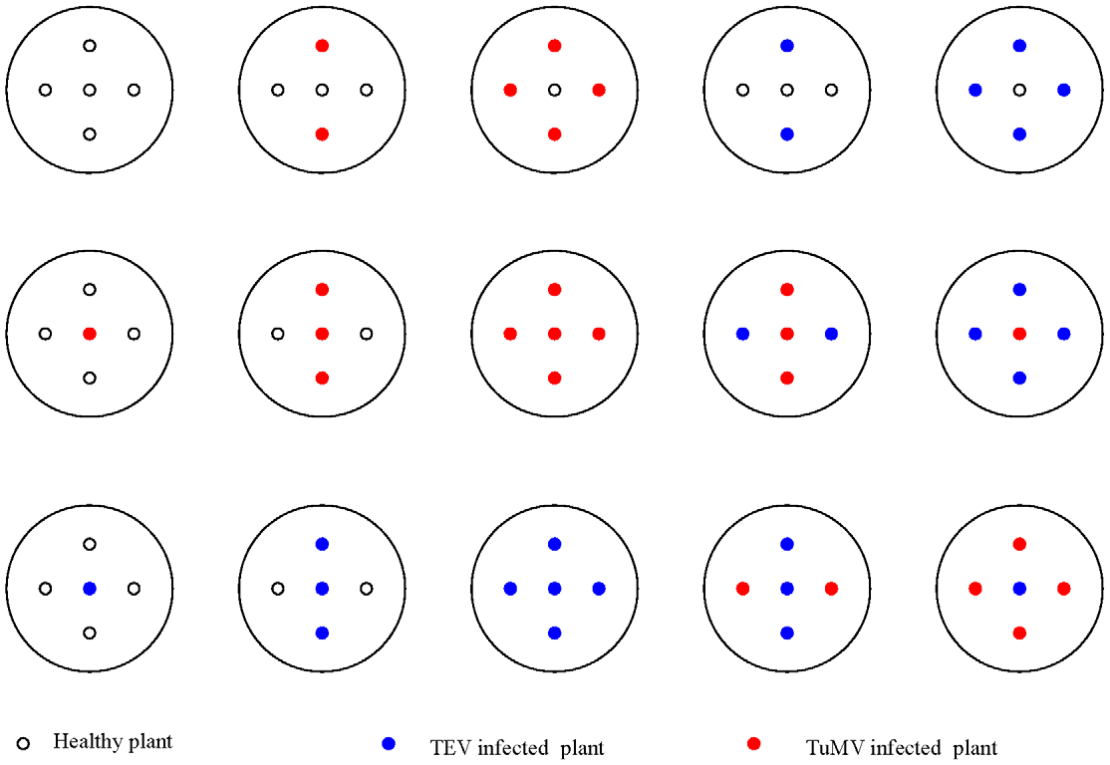
Experimental plan. The 15 combinations of healthy, TEV inoculated and TuMV inoculated plants in one experimental block. In each line, the central plant has the same infection status and the combinations have been ranked from high to low intraspecific competition (from left to right) as predicted from the known effects of the two viruses on *N. benthamiana* vegetative growth.

The experiment consisted in six blocks each constituted by 15 pots containing five *N. benthamiana* each (figure 2). A preliminary experiment established that five *N. benthamiana* in a 17 cm diameter pot present an important reduction of aerial part fresh weight compared to a single plant grown in the same pot. Five plants thus represent a condition where intraspecific competition plays an important role in shaping the growth pattern of the plant. In each pot, there was a central plant, which was the focal plant of the experiment. The four other plants, afterwards named “peripheric plants” were disposed at equal distance from the central one, forming a square around it. These plants are the competitors. The central plant and the peripheric ones were either inoculated with inoculation buffer (to produce healthy plants), or with a sap containing 150 mg of TuMV infected tissue homogenized in 255 µL of inoculation buffer or with a sap containing 150 mg of TEV infected tissue homogenized in 850 µL of inoculation buffer. This difference in the buffer volume allows correcting for the difference in LFU of the TEV and TuMV stocks. Each plant was mechanically inoculated 32 days after the seed was sawn with 5 µL of the sap on the third true leaf. Plants were maintained in the greenhouse at 25°C with 16 h of light.

The 15 different combinations represent 15 different situations of intraspecific competition because of the combination of infection status (healthy, TEV-infected and TuMV-infected) of the focal plant and the competitor plants. From the known symptoms of the two viruses, we could rank the combinations following the predicted intensity of competition exerted by the peripheric plants, as shown in figure 2.

12 dpi, the infection status of the plants was checked and the aerial parts of all the plants (focal and peripheric) were collected, measured (from the basis of the stem to the apex) and weighted to the nearest 10 mg. Moreover, the internode distances were measured on all the focal plants with a mechanical caliper and the mean of the internode distance in each plant was then used as a variable in statistical analyses.

All the statistical analyses were performed with JMP 7.0.1.

## Results

Over the 90 pots, 4 of them did not have the expected composition: three of them because one peripheric plant inoculated with TEV was not infected and one because one peripheric plant inoculated with TuMV was not infected. The data from these pots were not included in the analyses.

### Differences in virulence among TEV and TuMV

We first verified that the visual difference in symptoms between TEV and TuMV were confirmed by significant differences in height and fresh weight. For this, we used all the data (from central and peripheral plants) and performed a two-way ANOVA with infection (3 categories: healthy, TEV-infected and TuMV-infected) and block as factors and fresh weight and height as the dependent variables. Infection status was treated as a fixed factor whereas block was treated as a random factor. For both variables, infection had a significant effect (*F*_2,442_ = 1344.342, *P*<0.001; *F*_2,442_ = 487.363, *P*<0.001, respectively). TEV infected plants were 56.8% shorter and 59.2% lighter than healthy ones and TuMV infected plants were 56.5% shorter and 75.5% lighter than healthy ones. A Tukey HSD test indicated that TEV-infected and TuMV-infected plants significantly differed for fresh weight. This analysis confirmed that TuMV induced stronger symptoms than TEV on *N. benthamiana* and that this translated into a lower fresh biomass production for TuMV infected plants.

### The infection status of competitors has no direct effect on the focal plants

In order to determine if the growth of the central plant was affected by the infection status of its four competitors, we performed a two-way ANOVA with competitor status (5 levels) and block, for each of the three types of central plant (healthy, TEV-infected, TuMV-infected) separately. This corresponds to one analysis comparing the five situations in each of the three lines in figure 2. Block was taken as a random factor and the method of analysis was REML. These analyses were realized for the three variables: fresh weight, height and internode length. The full results are presented in table 1. There was no effect of the infection status of the competitors for any of the focal plants and any of the variables observed.

**Table 1:** Effect of the nature of competitors on plant growth.

		Nature of the competitors		Block
		*F* ratio	*P* value	% of variance explained
Healthy central plant	Plant fresh weight	0.809	0.536	3.25
	Plant height	1.790	0.176	47.62
	Internode length	1.075	0.399	25.57
TEV-infected central plant	Plant fresh weight	1.551	0.228	11.73
	Plant height	2.320	0.094	53.22
	Internode length	1.605	0.218	17.53
TuMV-infected central plant	Plant fresh weight	1.009	0.426	11.69
	Plant height	0.586	0.677	65.88
	Internode length	0.256	0.903	41.89

Results of two-way ANOVAs with nature of the competitors and block as effects for the three possible central plants.

One potential explanation to this lack of significance is that the competition exerted was actually not significantly different from one competitor composition to the other. To test this, we used the total fresh weight of competitor as a proxy for the intensity of competition and performed the same type of analyses as above with total fresh weight of competitor as dependent variable. The full results of this analysis are presented in table 2. The competitor composition has a very significant effect on the total fresh weight of competitor used as a proxy for the intensity of intraspecific competition. Moreover the predicted order of increasing intraspecific competition intensity was verified (see column “mean total competitor fresh weight” in table 2), except in one case (when the central plant is healthy, the sum of competitor fresh weight is higher for the “2 TuMV +2 healthy” category than for the “2 TEV +2 healthy” category, contrary to what was predicted). However, Tukey HSD tests reveal that in many cases the difference between the categories was not significant. In particular, the different competitor compositions usually fall in three significantly different categories: 0, 2 and 4 infected competitors. Said in another way, the differences in virulence of TEV and TuMV did not translate into differences in intensity of intraspecific competition, as far as the total fresh weight of competitor can reveal it. This is likely to be due to the high variability between plants reflected in the large standard errors.

**Table 2:** Comparison of the competitor total fresh weight for each central plant and competitor composition.

Central plant	Competitor categories	Mean total competitor fresh weight	Standard error of total competitor fresh weight.	Tukey HSD test.
Healthy*F*_4,17.02_ = 38.349*P*<0.001	4 TuMV	7.73	1.03	A
	4 TEV	14.40	1.49	A
	2 TuMV +2 healthy	22.46	2.70	B
	2 TEV +2 healthy	21.72	1.71	B
	4 healthy	32.22	3.31	C
TEV infected*F*_4,19.35_ = 22.787*P*<0.001	4 TuMV	6.80	0.97	A
	2 TEV +2 TuMV	10.50	0.88	A
	4 TEV	13.02	1.62	A
	2 healthy +2 TEV	24.85	4.93	B
	4 healthy	31.28	2.23	B
TuMV infected*F*_4,20_ = 51.240*P*<0.001	4 TuMV	8.03	1.30	A
	2 TEV +2 TuMV	10.18	0.51	A, B
	4 TEV	14.36	0.94	B
	2 healthy +2 TuMV	20.81	1.87	C
	4 healthy	33.80	3.17	D

The sum of competitor fresh weight is used as a proxy for the intensity of competition. Below each central plant is indicated the *F* and the *P* value corresponding to the effect of the factor “competitor composition” on the variable “total fresh weight of competitors”. The competitor categories are ordered for each central plant following the predicted increasing competition intensity. In the last column, categories not connected by the same letter (within each central plant category) are significantly different from each other in terms of total fresh weight of competitors.

With this data at hand, we decided to group the competitor compositions in the three categories revealed by the previous analysis, which is equivalent to using the prevalence of infected plants among competitors without taking into account if they are infected by TEV or TuMV. A three-way ANOVA was performed on the full data set with infection status of the central plant (3 categories: healthy, TEV-infected, TuMV-infected), prevalence among the competitors (3 levels: 0, 2 or 4 infected competitors) and block. The first two factors were taken as fixed factors and block as a random factor. The interaction between the two fixed factors was also included in the analysis. The method of analysis was REML. The dependent variables were plant height, plant fresh weight and average internode distance. The infection status of the central plant had a significant effect on the three variables (height: *F*_2,72_ = 414.694, *P*<0.001; fresh weight: *F*_2,72_ = 83.759, *P*<0.001; internode distance: *F*_2,69_ = 210.079, *P*<0.001), whereas the prevalence among the competitors and the interaction had no significant effect for any of the traits, reinforcing the idea that the intensity of intraspecific competition is not modified by parasitism in this pathosystem.

### Negative effect of infection on adaptive phenotypic plasticity

**Figure 3:**
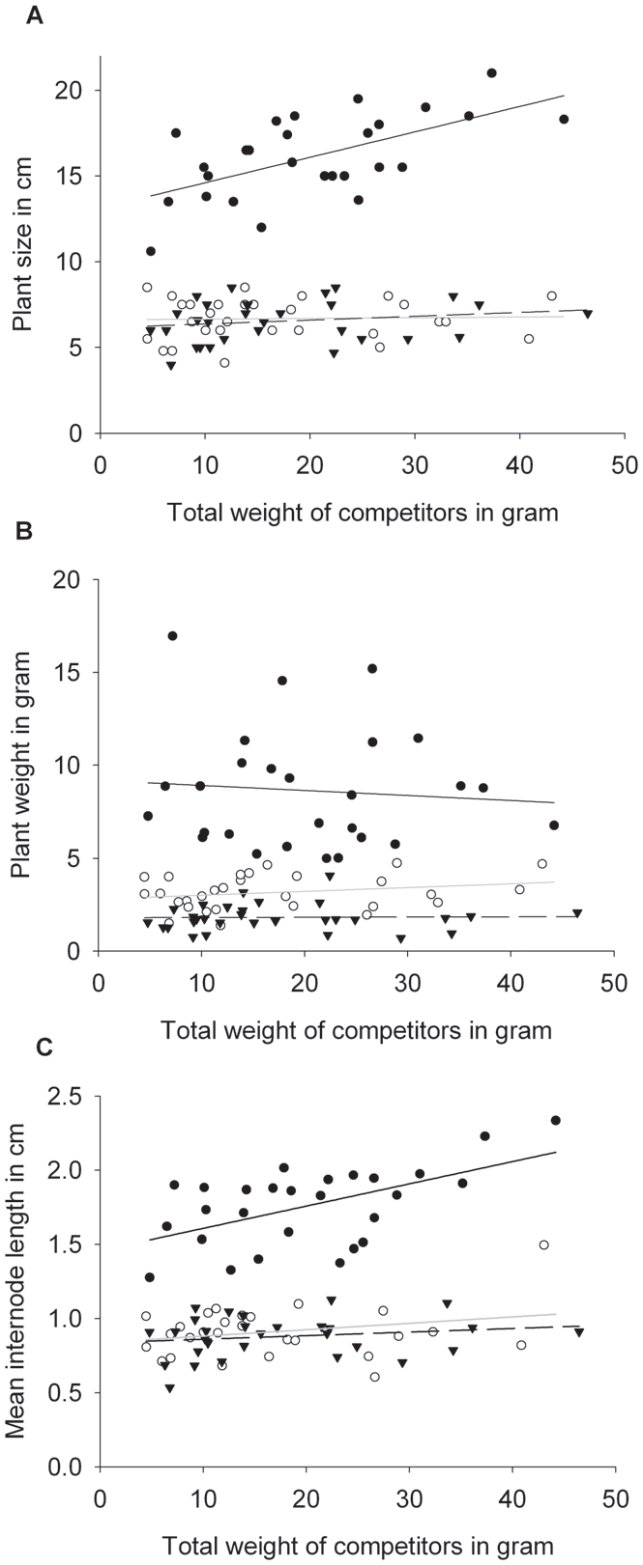
Effect of total competitor weight on plant growth. Regression of (*a*) height, (*b*) fresh weight and (*c*) medium internode length over total competitor fresh weight for healthy central plants (black circles, continuous line), TEV infected central plants (empty circles, grey line) and TuMV infected central plant (triangles, dashed line).

Finally, to determine if viral infection interplayed with the shade avoidance syndrome, we performed an ANCOVA with infection status of the central plant as a factor, the total fresh weight of competitors as a covariable and height, fresh weight and mean internode length of the central plant as dependent variables. The interaction between the fixed factor and the covariable was also included in the analysis. The full results of these analyses are presented in table 3. In brief, for fresh weight, the effect of the virus was significant but neither the total fresh weight of competitors nor the interaction term were significant. By contrast, for height and internode length, all effects, including the interaction, were significant. For these two variables, the interaction was due to the fact that for healthy plants, there is a positive relationship between the total fresh weight of competitors and the height or internode distance whereas this relationship does not exist for plants infected either by TEV or TuMV (figure 3). When looking at each focal plant independently, the regression between total competitor fresh weight and plant height is significant for healthy plants (*P*<0.001) but not for TEV-infected (*P* = 0.848) or TuMV-infected (*P* = 0.311) plants. In the same way, the regression between total competitor fresh weight and mean internode distance is significant for healthy plants (*P* = 0.003) but not for TEV-infected (*P* = 0.180) or TuMV-infected (*P* = 0.344) plants.

**Table 3:** Influence of total weight of competitor on plant growth.

Dependent variable	Factors	*F* ratio	*P* value
Height	Infection status of the focal plant	325.200	<0.001
	Total fresh weight of competitors	13.049	0.001
	Infection status × weight of competitors	6.944	0.002
Fresh weight	Infection status of the focal plant	89.982	<0.001
	Total fresh weight of competitors	0.001	0.981
	Infection status × weight of competitors	0.465	0.630
Internode distance	Infection status of the focal plant	188.214	<0.001
	Total fresh weight of competitors	13.689	<0.001
	Infection status × weight of competitors	3.803	0.027

ANCOVA with infectious status of the focal plant and total fresh weight of competitors as factors and either height, fresh weight or mean internode distance as dependent variable.

## Discussion

In the present pathosystem, we were able to detect neither a higher growth of healthy plants in competition with infected ones nor a higher virulence expressed by infected host in competition with healthy ones, neither for TEV nor for TuMV infections. These two trait modifications are characteristic indirect costs of parasitism due to the modification of the intraspecific competition relationship that has been identified in various other plant-pathogen systems and in a couple of animal-pathogen systems. This suggests that this phenomenon is not universal but depends on the peculiarities of each pathosystem. This absence of significant effect might be due to the absence of a real effect or to our inability to detect it. We might have missed the effect by measuring the variable too early or too late after infection: the indirect cost of parasitism might not be visible during early infection or early developmental stage of the plants as revealed in the *Chondrilla juncea-Puccinia chondrillina* system [9]. In this study, the interaction between intraspecific competition and infection by the rust was not detectable at the rosette stage but clear later in the plant development. This hypothesis seems, however, unlikely because at 12 dpi the difference between healthy, TEV-infected and TuMV-infected *N. benthamiana* was very clear and the growth pattern is markedly affected by the virus at this stage. Another possibility is that we missed the effect because of the high variance between plants, which makes the replicate number necessary to detect an effect very large.

Our results, however, strongly suggest the existence, in this system, of another indirect cost of parasitism. Indeed, we have shown that both the plants infected by TEV and by TuMV do not present the shade avoidance syndrome whereas the healthy plants have the characteristic increase in size and internode length at constant vegetative fresh weight when the intraspecific competition intensity is high. From the point of view of the host, since the shade avoidance syndrome is a case of adaptive phenotypic plasticity [6], our results indicate that a viral infection can be responsible for the loss of adaptive phenotypic plasticity, which is likely to result in a fitness decrease and therefore represents an indirect cost of parasitism.

From the point of view of the virus, the disappearance of the shade avoidance syndrome can be a side effect of virulence or could be adaptive: reducing the distance between the leaves is likely to provide a faster access to new leaves to colonize. Distinguishing between these two options represent an experimental challenge because it is difficult to manipulate plant growth so as to produce infected plants presenting a shade avoidance syndrome. At the mechanistic level, the virus might interact at any point between the photoreceptors of the plants and the reallocation of resources in consequence of the perception of the quality of the light in the environment.

Disruption of adaptive phenotypic plasticity by infection is a factor that has to be taken into account in the evaluation of the virulence of a pathogen and which will very likely play a role in the condition-dependent expression of virulence. Effects of pathogens on reaction norms to another environmental factors have rarely been studied as such but indirect information can be extracted from some studies. In the *Portulaca oleracea*–CMV pathosystem, healthy plants have decreasing reaction norms to the increase of plant density for a variety of reproductive and vegetative traits whereas plants infected with CMV have flat reaction norms, with all value in the reaction norms being below the ones of healthy plants (see figure 1 in ref. [12]). This represents another case of alteration of host phenotypic plasticity by a viral infection. However, in this case, the phenotypic plasticity in the absence of the pathogen is likely to result from a resource constraint and not to be adaptive (for a discussion on the adaptive value of phenotypic plasticity, see [19]). The mechanism behind the disappearance of phenotypic plasticity in this case is probably that in the presence of the pathogen, the resources from the environment are not the limiting factor anymore. The consequences in terms of fitness and virulence are also quite different: if the healthy host phenotypic plasticity is not adaptive, its suppression by the virus probably does not cause a decrease in host fitness.

Suppressing totally the phenotypic plasticity, as in the present study and as in Friess and Maillet [12] is an extreme case. The alteration of phenotypic plasticity can be subtler. For example, for the mosquito *Aedes aegypti*, an increasing proportion of larvae develop into pupae and then emerge as adults when the environmental resources increase. Infection by the microsporidiae *Vavraia culicis* triggers a shift of this emergence reaction norm to the right [20], which means that an infected population needs more resources to reach the same emergence rate than a healthy one. It has been further shown that this effect results from competition for resources between host and parasite [21]. In this example, the initial phenotypic plasticity is also non-adaptive and results from resource constraint, but because the pathogen does not suppress it - but shifts the reaction norm to the right - the effect of the pathogen on the phenotypic plasticity results in a host fitness reduction and is a component of virulence. Finally, in many studies [22]–[26], the effect of a pathogen has been studied in two, or more, different environmental conditions and a significant infection × condition interaction has been found, very often for the effect on mortality. This type of protocol does not directly address the effect of the pathogen on phenotypic plasticity but in some cases, the interaction might result from an alteration of the healthy host phenotypic plasticity by the pathogen.

To sum up, in the present study we could not find any evidence of indirect effect of parasitism through the modification of intraspecific competition but we showed the suppression of adaptive shade avoidance syndrome by the two potyviruses used. This suppression of adaptive phenotypic plasticity is likely to constitute a component of virulence, and to be one of the mechanisms explaining the condition-dependent expression of virulence in numerous systems. Establishing experimentally the importance of this virulence component will probably be challenging because of the difficulties of showing the adaptive value of phenotypic plasticity.

## References

[1] Stearns SC (1992). The Evolution of Life Histories..

[2] Santos M, Borash DJ, Joshi A, Bounlutay N, Mueller LD (1997). Density-dependent natural selection in Drosophila: Evolution of growth rate and body size.. Evolution.

[3] Borash DJ, Gibbs AG, Joshi A, Mueller LD (1998). A genetic polymorphism maintained by natural selection in a temporally varying environment.. Am Nat.

[4] Schmitt J, Dudley SA, Pigliucci M (1999). Manipulative approaches to testing adaptive plasticity: Phytochrome-mediated shade-avoidance responses in plants.. Am Nat.

[5] Franklin KA (2008). Shade avoidance.. New Phytol.

[6] Dudley SA, Schmitt J (1996). Testing the adaptive plasticity hypothesis: Density-dependent selection on manipulated stem length in *Impatiens capensis*.. Am Nat.

[7] Bedhomme S, Agnew P, Vital Y, Sidobre C, Michalakis Y (2005). Prevalence-dependent costs of parasite virulence.. Plos Biol.

[8] Finckh MR, Mundt CC (1996). Temporal dynamics of plant competition in genetically diverse wheat populations in the presence and absence of stripe rust.. J Appl Ecol.

[9] Burdon JJ, Groves RH, Kaye PE, Speer SS (1984). Competition in mixtures of suscptible and resistant genitypes of *Chondrilla juncea* differentially infected with rust.. Oecologia.

[10] Paul ND, Ayres PG (1986). Intereference between healthy and rusted groundsel (*Senecio vulgaris* L.) within mixed populations of different densitis and proportions.. New Phytol.

[11] Damgaard C, Jensen BD (2002). Disease resistance in Arabidopsis thaliana increases the competitive ability and the predicted probability of long-term ecological success under disease pressure.. Oikos.

[12] Friess N, Maillet J (1996). Influence of cucumber mosaic virus infection on the intraspecific competitive ability and fitness of purslane (*Portulaca oleracea*).. New Phytol.

[13] Pagan I, Alonso-Blanco C, Garcia-Arenal F (2009). Differential Tolerance to Direct and Indirect Density-Dependent Costs of Viral Infection in Arabidopsis thaliana..

[14] Sisterson MS, Averill AL (2003). Interactions between parasitized and unparasitized conspecifics: parasitoids modulate competitive dynamics.. Oecologia.

[15] Koprivnikar J, Forbes MR, Baker RL (2008). Larval amphibian growth and development under varying density: are parasitized individuals poor competitors?. Oecologia.

[16] Shukla DD, Ward CW, Brunt AA (1994). The *Potyviridae*..

[17] Carrasco P, de la Iglesia F, Elena SF (2007). Distribution of fitness and virulence effects caused by single-nucleotide substitutions in tobacco etch virus.. Journal of Virology.

[18] Kleczkowski A (1949). The transformation of local lesion counts for statistical analysis.. Ann Appl Biol.

[19] Gotthard K, Nylin S (1995). Adaptive plasticity and plasticity as an adaptation - a selective review of plasticity in animal morphology and life-history.. Oikos.

[20] Bedhomme S, Agnew P, Sidobre C, Michalakis Y (2004). Virulence reaction norms across a food gradient.. Proc Roy Soc Lond B.

[21] Rivero A, Agnew P, Bedhomme S, Sidobre C, Michalakis Y (2007). Resource depletion in *Aedes aegypti* mosquitoes infected by the microsporidia *Vavraia culicis*.. Parasitology.

[22] Jokela J, Lively CM, Taskinen J, Peters AD (1999). Effect of starvation on parasite-induced mortality in a freshwater snail (*Potamopyrgus antipodarum*).. Oecologia.

[23] Jokela J, Taskinen J, Mutikainen P, Kopp K (2005). Virulence of parasites in hosts under environmental stress: experiments with anoxia and starvation.. Oikos.

[24] Brown MJF, Loosli R, Schmid-Hempel P (2000). Condition-dependent expression of virulence in a trypanosome infecting bumblebees.. Oikos.

[25] Ferguson HM, Read AF (2002). Genetic and environmental determinants of malaria parasite virulence in mosquitoes.. Proc Roy Soc Lond B.

[26] Vale PF, Stjernman M, Little TJ (2008). Temperature-dependent costs of parasitism and maintenance of polymorphism under genotype-by-environment interactions.. J Evol Biol.

